# High levels of memory B cells are associated with response to a first tumor necrosis factor inhibitor in patients with rheumatoid arthritis in a longitudinal prospective study

**DOI:** 10.1186/ar4543

**Published:** 2014-04-15

**Authors:** Claire I Daien, Sarah Gailhac, Thibault Mura, Bernard Combe, Michael Hahne, Jacques Morel

**Affiliations:** 1 Department of Rheumatology, Lapeyronie Teaching Hospital, Av.Doyen Gaston Giraud, 34295, Montpellier, France; 2 Molecular Genetic Institut of Montpellier, CNRS, UMR5535, 1919 route de Mende, 34090 Montpellier, France; 3 Montpellier I university, 39 Rue Université, Montpellier, France; 4 Montpellier II university, 2 Place Eugène Bataillon, 34095 Montpellier, France; 5 Clinical investigation center, Saint Eloi Hospital, 80, avenue Augustin FLICHE, 34295 Montpellier, France; 6 Academic Medical Center, Meibergdreef 9, 1105 AZ, Amsterdam, the Netherlands

## Abstract

**Introduction:**

Tumor necrosis factor inhibitor (TNFi) therapy is effective for rheumatoid arthritis (RA). Some researchers have suggested that TNFi therapy affects B-cell homeostasis. We studied the effect of TNFi therapy on the distribution of peripheral B-cell subsets to elucidate B-cell–related biomarkers to predict the TNFi response.

**Methods:**

Peripheral B cells were analyzed for expression of CD19, CD27, CD38 and immunoglobulin D in 31 healthy donors and 96 RA patients, including 21 patients who were followed 3 months after TNFi initiation.

**Results:**

Treatment with steroids significantly altered the distribution of B-cell subsets. After we adjusted for age, sex and steroid dose, we found that patients with RA had B-cell subset proportions similar to controls. B-cell subset distributions did not differ upon use of TNFi at baseline or before or after TNFi introduction. TNFi responders (according to European League Against Rheumatism criteria) at 3 months had significantly higher proportions of CD27^+^ memory B cells at baseline, and ≥26% CD27^+^ cells at inclusion was associated with a relative risk of 4.9 (1.3 to 18.6) for response to TNFi treatment. CD27^+^ cells produced three times more TNFα than did TNFi-naïve B cells and were correlated with interferon γ produced from CD4^+^ cells in patients without TNFi treatment.

**Conclusions:**

In patients with RA, high levels of baseline memory B cells were associated with response to TNFi, which may be related to TNFα-dependent activation of the T helper type 1 cell pathway.

## Introduction

Rheumatoid arthritis (RA) is a common autoimmune disease with a prevalence of 0.3% to 1% worldwide. The disease is often associated with reduced mobility, increased social dependency and work-related disability [1]. RA is a systemic inflammatory disease affecting the joint-lining tissue, called the *synovium*. RA synovial tissue is characterized by increased numbers of macrophage-like and fibroblast-like synoviocytes in the intimal lining layer and infiltration of the sublining by macrophages, T and B cells, and other inflammatory cells that promote inflammation and destruction of bone and cartilage [2]. The intraarticular and systemic expression of proinflammatory cytokines—particularly tumor necrosis factor α (TNFα), interleukin 1 (IL-1) and IL-6, which are produced primarily by synovial macrophages and synoviocytes—plays a crucial role in the pathogenesis of RA, in that these cytokines can contribute to the increased number of the fibroblast-like synoviocytes.

TNFα is one of the most potent proinflammatory cytokines and is known to regulate cell survival, cell death and/or cell growth, depending on the cell type [3]. The blockage of TNFα with bioengineered compounds, either antibodies or soluble receptor molecules, can ameliorate the symptoms and joint destruction due to RA with remarkable efficacy. Two treatment strategies used to neutralize TNFα include the monoclonal antibodies infliximab and adalimumab; certolizumab pegol, a PEGylated antigen-binding fragment of a humanized antibody; and the soluble TNF receptor construct etanercept. The fusion protein etanercept consists of the extracellular ligand binding domain of TNF receptor 2 coupled to the Fc portion of a human antibody. Of note, because TNF receptors also bind the TNF ligand lymphotoxin α, etanercept antagonizes TNFα as well as lymphotoxin α.

TNF antagonists modulate disease development mainly by their anti-inflammatory properties. The multiple biological responses include reducing the production of proinflammatory cytokines, chemokines and acute-phase proteins; decreasing the expression of adhesion molecules; attenuating vascular permeability and angiogenesis; deactivating epithelial, endothelial and dendritic cells, as well as myofibroblasts and osteoclasts; increasing the number of circulating regulatory T cells; and diminishing the recruitment of inflammatory cells from blood to the inflamed tissue. Recently, TNF antagonists were suggested to regulate B-cell homeostasis [4,5].

B cells play a prominent role in RA because they produce rheumatoid factor (RF) and anticitrullinated peptide antibodies (ACPAs), which are well-established indicators of disease and disease severity and precede the onset of disease by many years. The pathogenic roles of these autoantibodies in RA was strongly suggested by the amplification of tissue injury by ACPAs in collagen-induced arthritis [6]. Moreover, B cells have antibody-independent functions that include cytokine secretion, antigen presentation and organization of other inflammatory cells. B cells play a crucial role in the development of tertiary lymphoid tissue within the inflamed synovium that seems to amplify autoimmune responses. B cells of the germinal center–like structures in the synovium are essential for CD4 T-cell activation. Depletion of B cells inhibits the T-cell production of interferon γ (IFN-γ), which is involved in the immune response, and of IL-1 in the rheumatoid synovium [7].

However, data concerning circulating B-cell subsets in RA are controversial [4,8,9]. Two studies focused on the possible effects of TNF inhibitor (TNFi) therapy on B cells, exploring whether such inhibition could help in the efficacy of B cells [4,5]. One study showed an increase in the proportion of preswitch memory B cells after TNFi therapy, and the other showed a decreased proportion of memory B cells in patients receiving TNFi therapy. The reasons for the differing findings are unclear, but they may be related to the cohort composition. An interesting difference between the two studies is the TNF antagonist used. Souto-Carneiro *et al*. used the anti-TNF monoclonal antibody infliximab [4], whereas Anolik and coworkers used the soluble TNF receptor fusion protein etanercept [5].

In our present study, we analyzed the effect of 3-month treatment with versus without TNFi therapy on the distribution of memory B cells in patients with active RA who were receiving synthetic disease-modifying antirheumatic drugs (DMARDs). We also compared the use of monoclonal antibody and soluble receptor TNFi therapy. We assessed baseline B-cell phenotypes associated with TNFi response and analyzed B-cell subset composition in a large cohort of RA patients and controls to assess the effect of RA characteristics on B-cell distribution.

## Methods

### Patients and controls

We enrolled consecutive patients with RA who met the 2010 American College of Rheumatology/European League Against Rheumatism (ACR/EULAR) criteria for RA [10]. Use of prednisone was permitted if doses were <10 mg/day and stable for at least 1 week, with the same requirement for synthetic DMARDs if doses were stable for at least 3 months. Patients who had previously received rituximab were excluded. All of these patients were included in the transversal study comparing RA patients with controls and patients naïve to TNFi treatment with those currently on TNFi therapy (regardless of the number of TNFi agents previously taken). Some patients were followed after the initiation of TNFi treatment. These patients were also recruited consecutively. A TNFi was introduced because of the presence of active disease (Disease Activity Score in 28 joints (DAS28) >3.2) despite treatment with synthetic DMARDs. For this longitudinal study, the inclusion criteria were, as for the transversal study, 2010 ACR/EULAR criteria fulfillment, prednisone doses <10 mg/day and stable for at least 1 week, and synthetic DMARDs and stable for at least 3 months. Moreover, these patients had to be naïve to any biological treatment. Controls were chosen on the basis of being in the same age range as the RA patients. They were either blood donors or patients seen in rheumatology departments for osteoarthritis, vertebral discopathy or other mechanical pain and free of any general pathology or infection.

Clinical and biological data were collected at baseline and at 3 months after TNFi therapy introduced at baseline. The DAS28 scores and concomitant treatments were evaluated at each time point. Response to treatment was defined on the basis of EULAR criteria [11]: a decrease in DAS28 score >0.6 between baseline and 3 months, with a DAS28 score <5.1 at 3 months. All patients gave their written informed consent to participate in the study, which was approved by the Medical Ethics Committee of Nimes University Hospital, France (2012-A00592-41).

### Flow cytometry

Peripheral blood mononuclear cells (PBMCs) were isolated from blood collected in ethylenediaminetetraacetic acid tubes by using of Ficoll reagent (Ficoll-Paque Plus; STEMCELL Technologies, Vancouver, BC, Canada). After gating lymphocytes using side scatter area/forward scatter (FSC) area and excluding doublets using FSC height/FSC width, B cells were phenotyped by flow cytometry (FACSCanto II cell analyzer; BD Biosciences, San Jose, CA, USA) with the antibodies BD Horizon V450-conjugated anti-CD19 (BD Biosciences), phycoerythrin (PE) anti–immunoglobulin D (anti-IgD) (BD Pharmingen, San Diego, CA, USA), PE/cyanine 7 (PE/Cy7) anti-CD38 (BD Pharmingen), and allophycocyanin anti-CD27 (BD Pharmingen). A minimum of 100,000 events in the lymphocyte gate were acquired. Memory B cells were defined as CD19^+^CD27^+^ lymphocytes with preswitch and postswitch memory B cells (CD27^+^IgD^+^ and CD27^+^IgD^-^) and double-negative memory B cells as CD27^-^IgD^-^. Naïve B cells were defined as CD19^+^CD27^-^IgD^+^ lymphocytes. The results are expressed as the percentage of positive cells. All analyses involved the use of fresh blood. We secondarily calculated the absolute number of cells using the routine complete blood count values when available.

Cells from some patients were frozen in fetal calf serum (FCS) with 10% dimethyl sulfoxide and kept at -80°C. Unfrozen cells were used to assess TNFα production from B-cell subsets and IFN-γ from CD4^+^ cells. For TNFα assessment, PBMCs were cultured in RPMI 1640 medium with 10% FCS for 4 hours with phorbol 12-myristate 13-acetate (0.1 μg/ml), ionomycin (0.5 μg/ml) and brefeldin A (10 μg/ml) (PIB). For CD4^+^ IFNg^+^ determination, PBMCs were cultured for 24 hours with anti-CD3 and anti-CD28 antibodies with PIB for the last 4 hours [12]. Activated cells were stained with anti-CD19, anti-CD27 and anti-IgD antibodies or with anti-CD19, anti-CD27 and anti-CD4 antibodies. Next, cells were permeabilized with BD Cytofix/Cytoperm buffer solution (BD Biosciences) and stained with PE–anti-TNF or PE/Cy7–anti-IFN-γ antibodies.

### Statistical analysis

Patient characteristics are described with percentages for categorical variables and mean ± SD or median (interquartile range) values for continuous variables. The distribution of continuous variables was tested by performing the Shapiro-Wilk test. Multivariate linear regression analysis was used to compare B-cell subset proportions adjusted for age, sex and steroid dose. The correlation of B-cell subsets and continuous variables was assessed by Spearman correlation. The variation in B-cell subset distribution between baseline and 3 months was assessed by performing a paired Student’s *t*-test or Wilcoxon signed-rank test. Comparison of B-cell distribution by TNFi type at 3 months involved a linear mixed model. This model included a subject-specific random intercept and fixed effects of time and groups (with and without adjustment for age, sex and steroid dose). Comparison of B-cell subset distributions between EULAR-defined responders and nonresponders was carried out using Student’s *t*-test or the Mann–Whitney *U* test. We determined a cutoff baseline level of B cells associated with EULAR response using receiver operating characteristic curve analysis and maximizing the Youden index (sensitivity + specificity - 1).

We anticipated that we would need a minimum sample size of eight patients to detect an increase of 3.5 ± 1.5% in CD27^+^ population between baseline and 3 months, as previously reported by Souto-Carneiro *et al*. [4], with a power of 90% and an α value of 0.05 (considering a null intraindividual correlation). Statistical analyses were carried out using SAS version 9.3 software (SAS Institute, Cary, NC, USA). Two-tailed *P*-values <0.05 were considered statistically significant.

## Results

### Patients

To assess B-cell distribution in RA, we included 96 patients with RA and 31 controls (71% and 68% females, respectively, with mean age ± SD = 59 ± 13 years and 51 ± 16 years, respectively). For assessment of the TNFi effect, we included 21 patients (75% female, median age = 57 years (IQR == 50 to 62), median RA duration 7 years (IQR = 2 to 28)). Eleven patients received etanercept and ten were given monoclonal antibodies (one was given adalimumab and nine received certolizumab pegol) (Table 1).

**Table 1:** **Patient and control characteristics at baseline**^**a**^

**Characteristics at time of inclusion**	**Controls**	**All RA patients**	**DMARD-naïve patients**	**TNFi-naïve patients**	**TNFi ongoing**	**Baseline TNFi introduction**
	**(*N*** **= 31)**	**(*N*** **= 96)**	**(*N*** **= 18)**	**(*N*** **= 58)**	**(*N*** **= 21)**	**(*N*** **= 21)**
Monoclonal antibodies/soluble receptors (*n*)	–	–	–	–	12/9	10/11
Females (%)	68	71	78	71	76	75
Age, yr (mean ± SD)	51 ± 16	59 ± 13	59 ± 16	58 ± 14	59 ± 11	59 ± 11
RA duration, yr (median (IQR))	–	10 (4 to 21)	4 (3 to 19)	8 (3 to 22)	9 (5 to 16)	7 (2 to 28)
RF positivity (%)	–	76	76	68	80	81
ACPA positivity (%)	–	76	76	71	74	71
RF- and ACPA-negative (%)		21	22	21	19	19
Radiographic erosions (%)	–	73	65	67	81	71
Ongoing synthetic DMARD (%)	–	69	0	46	71	77
Previous TNFi (%)	–	36	0	0	86	0
Previous use of other biologic drugs (*n*)	–	40	0	0	19	0
Use of steroids (<10 mg/day) (%)	0	60.4	50.0	53.3	66.6	54.5
Current steroid dose (mg/day)	0	5 (0 to 9)	2.5 (0 to 7.8)	2.5 (0 to 7.6)	5.5 (0 to 8.6)	5 (0 to 9.2)
C-reactive protein level (mg/dl)	–	1.1 (0.4 to 2.4)	0.8 (0.3 to 2.8)	0.9 (0.4 to 1.9)	1.9 (0.8 to 2.8)	1.4 (0.4 to 2.6)
DAS28 score (median (IQR))	–	4.2 (3.4 to 5.5)	4.2 (3.4 to 5.0)	3.9 (3.3 to 4.7)	5.7 (5.1 to 6.4)	4.5 (2.8 to 5.0)

^a^ACPA, Anticitrullinated peptide antibody; DAS28, Disease Activity Score in 28 joints; DMARD, Disease-modifying antirheumatic drug; RA, Rheumatoid arthritis; RF, Rheumatoid factor; TNFi, Tumor necrosis factor inhibitor. DMARD-naïve patients and patients at baseline of TNFi introduction are included in the TNFi-naïve patient group. TNFi ongoing patients are different from all the other subgroups. All analyses are adjusted for age, sex and glucocorticoid dose.

### Effect of glucocorticoid treatment on B-cell composition

Sex significantly influenced the composition of CD27^+^IgD^-^ postswitch memory, CD27^-^IgD^+^ naïve and CD27^-^IgD^-^ B cells in controls (with, respectively, 10.9 (5.9 to 13.5), 76.7 (73.1 to 83.4) and 2.2 (1.7 to 2.5) in men and 20.9 (12.4 to 27.9), 65.7 (49.9 to 76.4) and 4.4 (2.8 to 7.4) in women; all *P* < 0.05), but not in RA patients. In controls, age was positively correlated with the proportion of CD27^-^IgD^-^ B cells (*r* = 0.42, *P* = 0.02), whereas age in RA patients was inversely correlated with only CD27^+^IgD^+^ preswitch memory B cells (*r* = -0.19, *P* = 0.04). Administration of steroids affected the distribution of B-cell subsets. Steroid dose was positively correlated with the proportion of memory B cells (*r* = 0.25, *P* = 0.008) and inversely with that of naïve B cells (*r* = -0.23, *P* = 0.02) (Table 2). Steroid users had greater proportions of memory B cells, especially postswitch memory B cells, and lesser proportions of naïve B cells than nonusers (*P* = 0.01, *P* 0.02 and *P* 0.006, respectively). These results strongly support the need to take into account steroid treatment when comparing controls and RA patients. After adjustment for age, sex and steroid dose, B-cell composition did not differ between RA patients and controls (Table 3), between controls and never-treated patients with RA, or between controls and patients with active RA (DAS28 score ≥3.2). In terms of absolute values, there was a global B-cell lymphopenia in RA patients (Additional file 1).

**Table 2:** **Correlation of rheumatoid arthritis characteristics and B-cell subset distributions**^**a**^

	**Lymphocytes (%)**	**B cells (%)**				
**Characteristics**	**CD19**^**+**^	**CD27**^**+**^	**CD27**^**+**^**IgD**^**+**^	**CD27**^**+**^**IgD**^**-**^	**CD27**^**-**^**IgD**^**+**^	**CD27**^**-**^**IgD**^**-**^
RA duration, yr	-0.23^b^	-0.11	-0.10	-0.13	0.07	0.08
Steroid dose, mg/day	-0.06	0.25^c^	0.20	0.27^c^	-0.23^b^	0.16
TNFi agents used (*n*)	-0.16	-0.15	-0.07	-0.17	0.16	-0.21^b^
DAS28 score	0.03	0.10^c^	0.18^c^	0.02	-0.10	-0.04^c^

^a^DAS28, Disease Activity Score in 28 joints; Ig, Immunoglobulin; RA, Rheumatoid arthritis; TNFi, Tumor necrosis factor inhibitor. ^b^*P* < 0.05, ^c^*P* < 0.01. ^c^Correlations were not significant; however CD27^+^, CD27^+^IgD^+^ and CD27^-^IgD^-^ proportions were higher in patients with active RA than in those with low disease activity after adjustment for age, sex and steroid dose (general linear models). Data are Spearman’s *r* statistics.

**Table 3:** **Distribution of B-cell subsets in patients and controls**^**a**^

**B-cell subsets**	**Controls**	**All RA patients**	**DMARD-naïve patients**	**p1**	**p2**	**TNFi-naïve patients**	**TNFi ongoing**	**p3**	**Baseline TNFi introduction**		**p4**
									**Baseline**	**3 months**	
CD19^+^	6.8 (2.5 to 8.7)	4.4 (3.3 to 6.1)	4.1 (3.1 to 9.6)	NS	NS	4.8 (3.6 to 7.4)	4.4 (3.1 to 6.3)	NS	5.3 (3.9 to 6.3)	7.7 (6.7 to 10.6)	**
(% lymphocytes)											
CD27^+^	22.0 (18.7 to 34.8)	25.4 (16.8 to 37.6)	34.4 (17.6 to 44.4)	NS	NS	25.2 (17.7 to 36.4)	30.0 (11.7 to 42.7)	NS	28.3 (19.6 to 36.2)	28.4 (19.0 to 39.6)	NS
(% CD19^+^)											
CD27^+^IgD^+^	10.4 (6.2 to 15.5)	8.0 (4.6 to 13.2)	8.0 (4.3 to 10.0)	NS	NS	8.0 (4.9 to 12.9)	10.5 (4.1 to 15.2)	NS	9.3 (5.4 to 14.2)	7.5 (3.4 to 12.7)	NS
(% CD19^+^)											
CD27^+^IgD^-^	15.4 (10.2 to 21.7)	16.6 (11.0 to 25.3)	22.2 (13.8 to 39.1)	NS	NS	15.2 (10.7 to 24.4)	17.3 (9.2 to 28.6)	NS	15.9 (12.7 to 24.5)	21.3 (13.2 to 24.8)	NS
(% CD19^+^)											
CD27^-^IgD^+^	73.1 (58.2 to 77.1)	65.7 (54.2 to 77.1)	58.5 (45.4 to 74.8)	NS	NS	68.5 (56.8 to 77.0)	65.0 (50.9 to 82.1)	NS	63.5 (54.4 to 76.7)	62.1 (49.6 to 73.7)	NS
(% CD19^+^)											
CD27^-^IgD^-^	2.8 (1.9 to 4.5)	4.7 (3.0 to 7.2)	5.8 (3.2 to 9.5)	NS	NS	4.7 (3.0 to 6.7)	3.8 (2.9 to 7.5)	NS	4.7 (3.0 to 6.9)	6.8 (4.2 to 10.3)	NS
(% CD19^+^)											

^a^DMARD, Disease-modifying antirheumatic drug; Ig, Immunoglobulin; NS, Not significant; p1, *P*-value comparing controls and all RA patients; p2, *P*-value comparing controls and DMARD-naïve patients; p3, *P*-value comparing TNFi-naïve and TNFi ongoing (currently taking TNFi agent); p4, *P*-value comparing baseline and 3-month data for patients with TNFi introduced at baseline; RA, Rheumatoid arthritis; TNFi, Tumor necrosis factor inhibitor. CD27^+^ memory B cells, CD27^+^IgD^+^ preswitch memory B cells, CD27^+^IgD^-^ postswitch memory B cells, CD27^-^IgD^+^ naïve B cells, CD27^-^IgD^-^ double-negative B cells, CD38^high^ plasmablasts. All values are expressed in median (IQR). P-values were adjusted for age, sex and steroid dose.

### Effect of rheumatoid arthritis characteristics and treatment on B-cell subset distribution

RA duration was inversely correlated with proportion of B cells (CD19^+^) among lymphocytes (*r* = -0.23, *P* = 0.02), but not B-cell subset distribution. The number of previous TNFi agents used was inversely correlated with proportion of CD27^-^IgD^-^ B cells (*r* = -0.21; *P* = 0.03 and *P* = 0.01 after adjustment for steroid dose) (Table 2). After adjustment for age, sex and steroid dose, as compared with patients with low disease activity (DAS28 score <3.2), patients with active disease showed greater proportions of CD27^-^IgD^-^ B cells (5.6 ± 3.9 vs 4.9 ± 2.6; *P* = 0.008) and memory B cells (27.7 ± 14.4 vs. 19.5 ± 10.7; *P* = 0.05), especially preswitch memory B cells (10.2 ± 7.5 vs. 5.3 ± 3.3; *P* = 0.04). No difference was found in the distribution of B-cell subsets between patients with versus without RF after adjustment for age, sex and steroid dose.

### Effect of tumor necrosis factor inhibitor therapy on B-cell distribution

After adjustment for age, sex and steroid dose, TNFi-naïve patients (*n* = 58) and those receiving TNFi therapy at baseline (*n* = 21) did not differ in B-cell subset distribution. Among patients receiving TNFi therapy at baseline, nine received etanercept and twelve were given monoclonal antibodies. Patients who received etanercept and monoclonal antibodies did not differ in B-cell subset distribution, nor did TNFi-naïve patients or patients receiving the two TNFi types. The number of B cells significantly increased 3 months after TNFi initiation, from a median of 5.3% (IQR 3.9 to 6.3) to 7.7% (6.7 to 10.6) (*P* < 0.01), with no change in B-cell subset distribution (Table 3). The change in median proportions of CD27^-^IgD^-^ B cells differed with etanercept and monoclonal antibody treatment (+1.6% (0.0 to 5.4) vs. 0.3% (-1.3 to 1.8) of B cells; *P* = 0.02). After adjustment for age, sex and steroid dose, however, this finding was no longer significant (*P* = 0.09). In terms of absolute values, there was no significant change between TNFi-naïve and TNFi-ongoing patients nor between baseline and 3 months after TNFi introduction (Additional file 1).

### Baseline B-cell phenotypes as predictors of tumor necrosis factor inhibitor response

**Figure 1:**
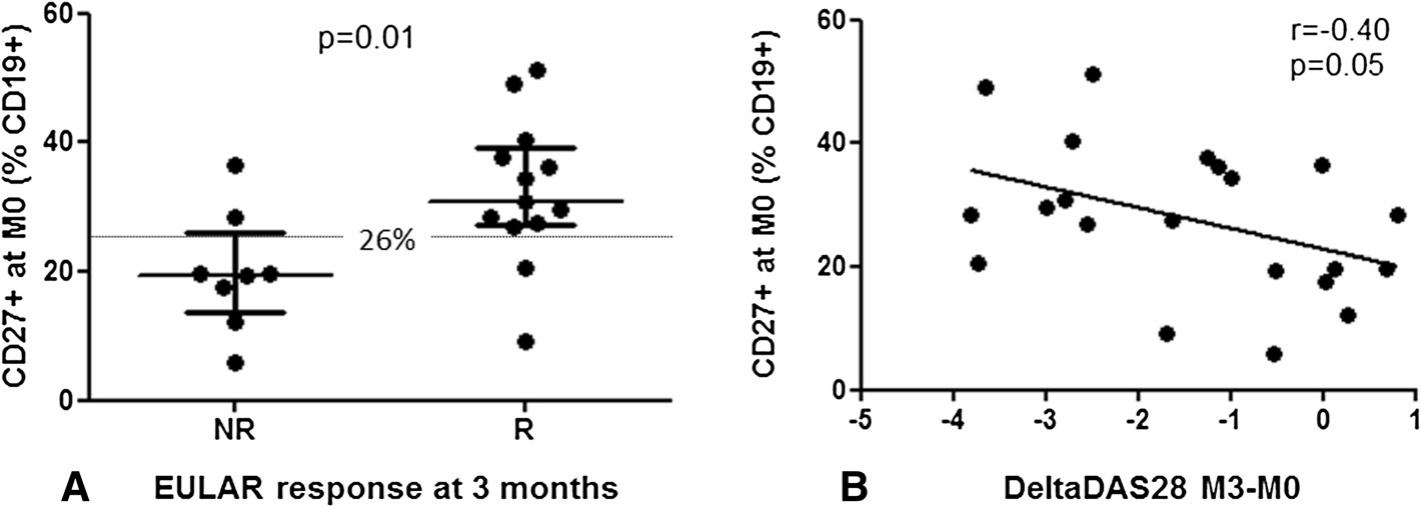
**Proportion of CD27**^**+ **^**memory B cells predicts response to tumor necrosis factor inhibitor therapy. (A)** Patients with response (R) or no response (NR) (according to European League Against Rheumatism (EULAR) criteria) to tumor necrosis factor inhibitor (TNFi) at baseline (M0) (*P* = 0.01). The gray line indicates 26% of memory B cells, the best threshold at which to separate responders and nonresponders. **(B)** Levels of CD27^+^ B cells at baseline correlated with change in Disease Activity Score in 28 joints (DeltaDAS28) during the first 3 months of TNFi treatment (*P* = 0.05). Each data point represents one participant. Horizontal bars are medians, and whiskers are IQR (25th to 75th percentile).

We compared baseline proportions of B-cell subsets among EULAR criteria–defined responders and nonresponders at 3 months. Baseline B-cell subset proportion was correlated with DAS28 score in the first 3 months of treatment, with significant differences for CD27^+^ memory B cells (Figure 1). CD27^+^ B-cell proportion at baseline was greater for EULAR criteria–defined responders than nonresponders at 3 months (30.8% (27.2 to 39.1) vs. 19.6% (13.6 to 26.1) of CD19^+^ cells; *P* = 0.01) (Figure 1). Baseline CD27^+^ B-cell proportion was inversely correlated with DAS28 score at 3 months (*r* = -0.40, *P* = 0.05). Notably, CD27^+^ memory B-cell proportion at baseline was not correlated with DAS28 score at the time of inclusion and was similar for patients with and those without RF. Responders and nonresponders had received similar doses of prednisone at baseline.

### Determination of predictive baseline threshold of CD27^+^ B cells

**Figure 2:**
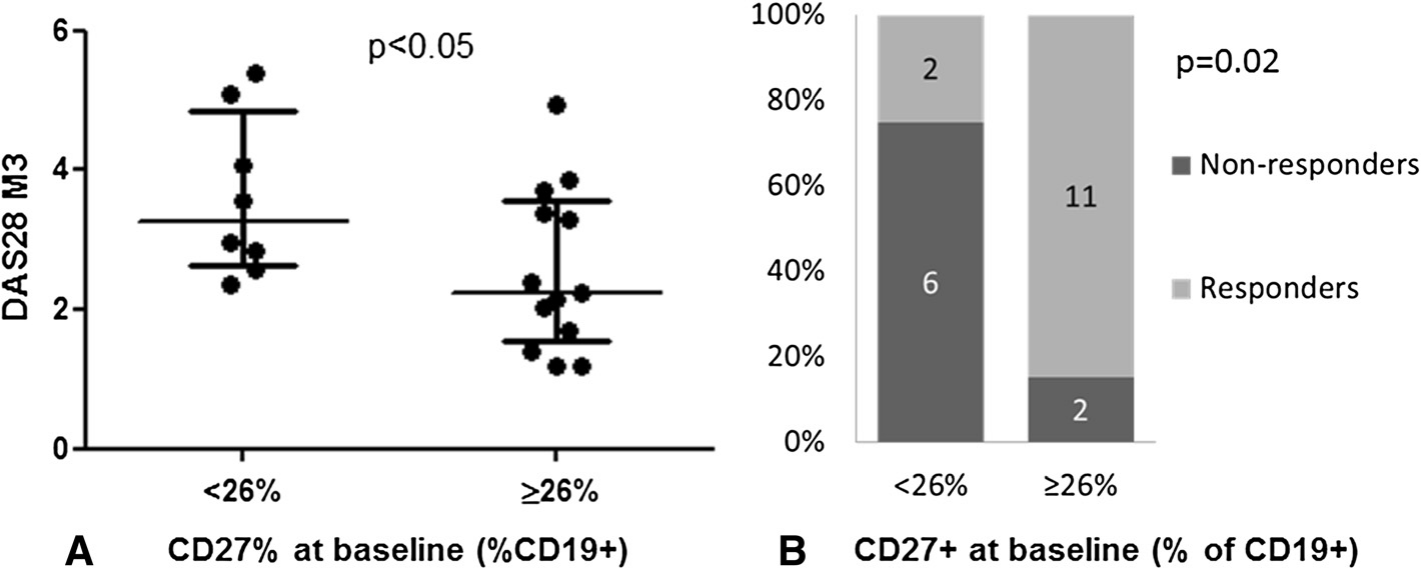
**Threshold of 26% for CD27**^**+ **^**B cells as a predictor of tumor necrosis factor inhibitor response. (A)** Association of CD27^+^ B-cell proportion ≥26% and <26% at baseline and Disease Activity Score in 28 joints (DAS28) after 3 months of tumor necrosis factor inhibitor (TNFi) therapy (*P* = 0.01). **(B)** Association of CD27^+^ B-cell proportion ≥26% at baseline and European League Against Rheumatism response (4.9 (1.3 to 18.6); *P* = 0.02). Each data point represents one participant. Horizontal bars are medians, and whiskers are IQR (25th to 75th percentile).

To further explore the use of CD27^+^ B-cell proportion for prognosis, we defined a threshold proportion of 26% (sensitivity = 92%, specificity = 71%, Youden index = 63%, positive predictive value = 85% and negative predictive value = 83%). For patients with ≥26% CD27^+^ B cells at baseline, DAS28 score was lower at 3 months (*P* < 0.05) and the relative risk of response was 4.9 (95% confidence interval = 1.3 to 18.6) (Figure 2). Of note, patients with CD27^+^ B cells ≥26% or <26% had received similar doses of steroids at baseline.

### Predictive value of CD27^+^ B cells assessed by flow cytometry

**Figure 3:**
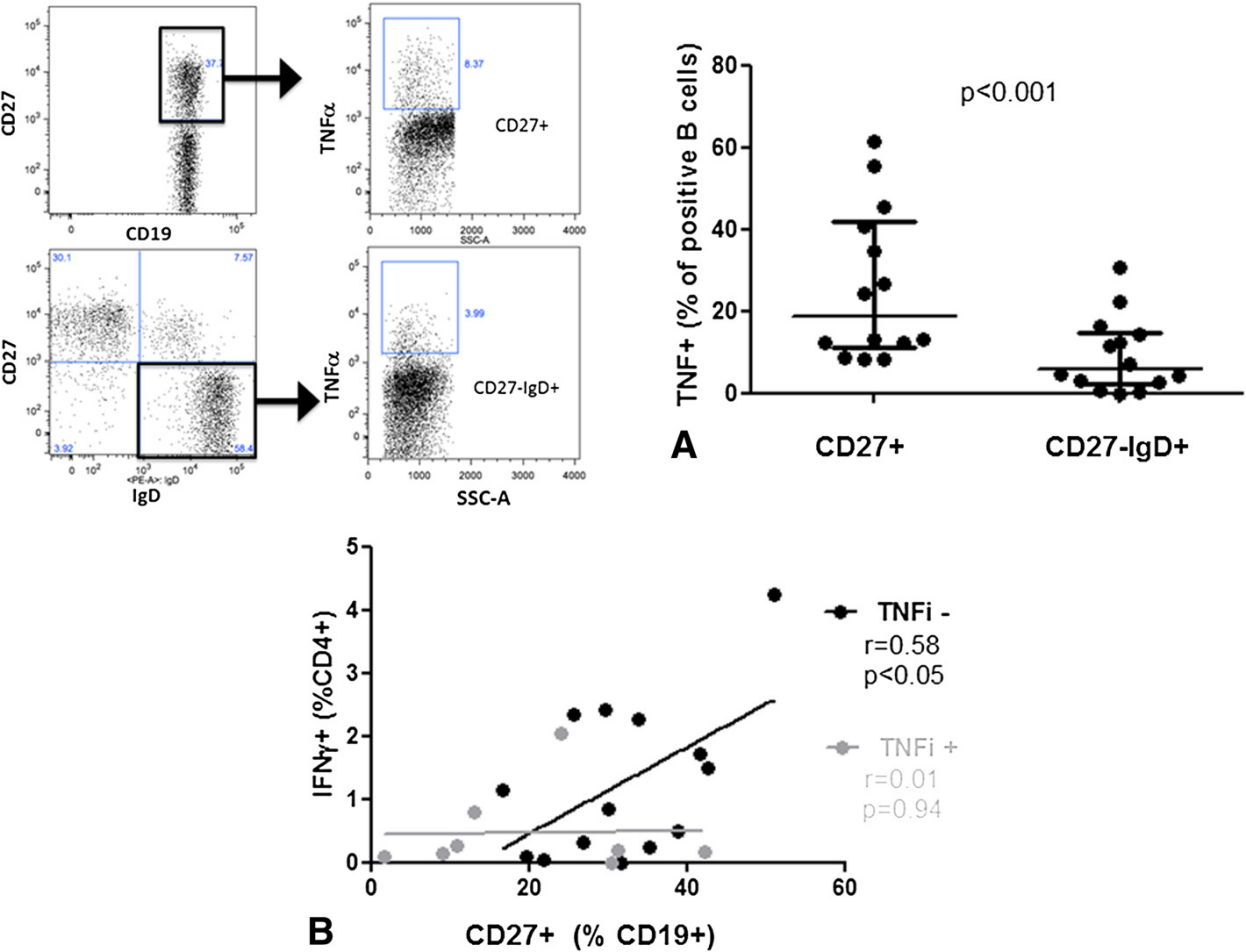
**CD27**^**+ **^**memory B cells were greater producers of tumor necrosis factor α than naïve B cells and inversely correlated with interferon γ–producing CD4**^**+ **^**cells in tumor necrosis factor–naïve patients. (A)** Flow cytometry of tumor necrosis factor α (TNFα) production by CD27^+^ memory B cells and naïve CD27^-^IgD^+^ B cells in 14 rheumatoid arthritis (RA) patients at baseline (*P* < 0.001). Each data point represents one participant. Horizontal bars are medians, and whiskers are IQR (25th to 75 percentile). IgD, Immunoglobulin D; SSC-A, Side-scatter area **(B)** Correlation of proportion of CD27^+^ memory B cells and interferon γ (IFN-γ)–producing CD4^+^ cells in patients without therapy (TNFi^-^, *n* = 14) and with therapy (TNFi^+^, *n* = 8).

We next analyzed the mechanisms underlying the predictive value of CD27^+^ B cells and assessed intracellular TNFα levels by flow cytometry. TNFα production was greater by threefold from CD27^+^ B cells than naïve B cells (18.8% (11.3 to 41.7) vs. 5.9% (2.3 to 14.7) of positive CD19^+^ cells; *P* < 0.001) (Figure 3A). Production of TNFα was greater from CD27^+^ memory B cells than from treatment-naïve B cells, and CD27^+^ memory B cells were inversely correlated with IFN-γ-producing CD4^+^ cells in TNFi-naïve patients.

The results of previous work have suggested that B cells can activate IFN-γ production in CD4^+^ T cells via TNFα. We therefore correlated the proportion of CD4^+^IFN-γ^+^ cells in patients with and without TNFi (*n* = 8 vs. *n* = 14) with that of CD27^+^ memory B-cell levels. In patients not receiving TNFi, the proportion of CD27^+^ B cells was positively correlated with that of CD4^+^IFN-γ^+^ cells (*r* = 0.58, *P* < 0.05), but not in patients receiving TNFi (*r* = 0.01, *P* = 0.94) (Figure 3B).

## Discussion

In the present study, we found B-cell lymphopenia in RA patients, with a similar distribution of naïve and memory B cells between RA patients and controls. Proportions of CD27^-^IgD^-^, CD27^+^ and CD27^+^IgD^+^ memory B cells were higher in patients with active than in those with low-level disease. TNFi therapy globally increased CD19^+^ B-cell proportions without modifying B-cell subset proportions. Of note, a high proportion of CD27^+^ memory B cells at baseline was associated with good clinical response to TNFi.

Although B cells are considered to have an important role in RA, data concerning B-cell subsets in the peripheral blood of patients with RA remain controversial. Souto-Carneiro *et al*. described a decreased proportion of CD27^+^IgD^+^ preswitch memory B cells in patients with RA and an increased proportion of CD27^+^IgD^-^ postswitch memory B cells in patients with long-term RA versus short-term RA and controls [4]. Anolik *et al*. did not find any change in the proportion of B-cell subsets in patients with RA [8]. de la Torre *et al*. found an increased proportion of only double-negative B cells [13]. Tony *et al*. found an increased proportion of CD27^+^ cells in RA patients positive for RF versus RA patients negative for RF and controls [9]. In a large cohort of 208 patients with RA, Sellam *et al*. found a decreased proportion of CD19^+^ total B cells and CD27^+^IgD^-^ B cells in RA patients, but no differences in proportions of global memory B cells or naïve B cells [14]. Defining memory B cells using only IgD and CD27 staining is generally accepted, but other markers could be used. Indeed, CD27^+^IgD^-^ postswitch memory B cells are mainly IgG^+^ or IgA^+^, but they can also contain a small fraction of IgM-only memory cells. Memory B cells can also be assessed using a Bm1 to Bm5 classification, with memory B cells being CD38^+^IgD^-^ and CD38^-^IgD^-^ (early and late Bm5, respectively). We might have missed differences between the studied groups because of the lack of Bm1 to Bm5 and IgM, IgG and IgA assessment.

We found that age and sex as well as steroid dose were important confounding factors that must be taken into account when comparing RA patients and controls, even at low steroid doses (<10 mg/day). Adjusted analyses eliminated biases, which could explain the contradictory results of other studies. After adjusting our analyses on these three variables, we found no differences in the proportions of all B-cell subsets between controls and all RA patients or never-treated RA patients. Proportions of CD27^-^IgD^-^, CD27^+^ and CD27^+^IgD^+^ memory B cells were greater in patients with active rather than low disease activity. These subsets are all memory B cells, as suggested by previous work on double-negative B cells [15,16]. Memory B cells accumulate in the synovium [17]. Because analysis of B cells in the peripheral blood is affected by B cells in the joint compartment, the increased production of memory B cells in the peripheral blood of RA patients may be compensated by joint migration. With active disease, however, the increase in the proportion of memory B cells could concern both peripheral blood and the joint compartment.

Data concerning the effect of TNFi therapy on B cells are also controversial. In a cross-sectional study, Anolik *et al*. found decreased proportions of circulating total CD27^+^ memory B cells in patients with RA receiving etanercept (*n* = 34) compared with patients receiving methotrexate (*n* = 17) and healthy controls [5]. Sellam *et al*. found an increased proportion of CD19^+^ and a decreased proportion of CD27^-^IgD^-^ B cells in patients receiving methotrexate and TNFi (*n* = 142) compared with patients receiving methotrexate alone (*n* = 66) [14]. These results were not adjusted for age, sex or steroid dose. In longitudinal studies, Souto-Carneiro *et al*. found an increased proportion of CD27^+^IgD^+^ B cells after infliximab treatment in 15 patients with RA [4], whereas Roll *et al*. found an increased proportion of CD27^+^ B cells after TNFi in RF-negative RA patients, but not in RF-positive RA patients [9].

In the present study, analyses adjusted for age, sex and steroid dose revealed a similar B-cell distribution in patients receiving TNFi and TNFi-naïve patients. We confirmed the increase in total CD19^+^ proportion found by Sellam *et al*. [9], but we did not find any significant changes in proportions of B-cell subset distributions after the introduction of TNFi. We could not assess the influence of RF positivity on changes in B-cell subset proportions, because our sample included only four RF-negative patients receiving TNFi. The data did not differ between use of monoclonal antibodies and the soluble receptor etanercept. Therefore, TNFi seems to globally increase the proportion of all B-cell subsets with respect to their normal distribution in peripheral blood.

As mentioned previously, TNFi therapy does not modify the proportion of B-cell subsets in peripheral blood. To explain the predictive value of memory B cells, their function should be altered by TNFi. In fact, we show that memory B cells could produce three times more TNFα than could naïve B cells. B cells are an important source of TNFα, as shown by other previous studies, as well as being the main producer of lymphotoxin α [18,19]. This finding could explain why baseline memory B cell levels affected the TNFi response. However, other sources of TNFα are more abundant, such as macrophages. Menard *et al*. reported that IFN-γ expression in splenic host T cells in B-cell–deficient mice was greater with B-cell transfer from infected than naïve mice (19). This IFN-γ production was mediated by the TNFα and cellular contact between B and CD4^+^ cells, as shown by the lack of efficacy of TNFα^-/-B^-cell transfer and by culturing CD4^+^ T and B cells separately in transwell plates [20]. Indeed, B cells amplified IFN-γ production by T cells via a TNFα-mediated mechanism. To further explore this hypothesis, we examined the relationship between memory B cells and CD4^+^IFN-γ^+^ T helper type 1 (Th1) cells. The proportion of CD27^+^ B cells and CD4^+^IFN-γ^+^ Th1 cells was correlated in patients free of TNFi, but not in patients currently taking TNFi. This indirect argument might suggest the importance of TNFα produced by B cells to induce the Th1 pathway in a microenvironment. Studies aimed at better exploring the relation between memory B cells produced in the TNFα and Th1 pathways are required.

The results of our present study are particularly interesting in the context of studies of patients receiving rituximab therapy. Other research groups have found a high proportion of baseline CD27^+^ memory B cells associated with poor clinical response and early relapse with rituximab therapy [14,21], whereas we found an association of TNFi therapy with good clinical response. Notably, in the work by Sellam *et al*. [9], the mean baseline CD27^+^ proportion was 26.8% for rituximab responders and 35.9% for nonresponders. CD27^+^ <26% at baseline may be a valuable threshold to distinguish TNFi nonresponders who may respond better to rituximab therapy. However, this threshold needs to be confirmed in other cohorts receiving TNFi and rituximab. Moreover, further studies are needed to address whether CD27^+^ memory B-cell levels can predict response to a second or a third TNFi therapy.

## Conclusion

The distribution of B-cell subsets in blood is influenced by age, sex and glucocorticoid dose, but it does not differ between patients with RA and controls. Patients with active RA have an increased proportion of memory B cells. Among the B-cell subsets, memory B cells are the most important TNFα producers, and this production is correlated with Th1 activation. Use of a TNFi appears to inhibit TNFα-mediated interaction between B and CD4^+^ cells, thus leading to decreased IFN-γ production by CD4^+^ T cells. High baseline levels of CD27^+^ memory B cells was found to be associated with a EULAR-criteria defined response to TNFi therapy, along with a 4.9-fold increased chance of response among patients with CD27^+^ levels ≥26%. Although further validation is needed, these findings might become an important tool in clinical practice.

## Abbreviations

ACPA: Anticitrullinated peptide antibody; ACR/EULAR: American College of Rheumatology/European League Against Rheumatism; BFA: Brefeldin A; DAS28: Disease Activity Score in 28 joints; DMARD: Disease-modifying antirheumatic drug; FCS: Fetal calf serum; IFN-γ: Interferon γ; IL: Interleukin; PBMC: Peripheral blood mononuclear cell; PIB: Phorbol 12-myristate 13-acetate, ionomycin and brefeldin A; RA: Rheumatoid arthritis; RF: Rheumatoid factor; SD: Standard deviation; Th1: T helper type 1 cell; TNF: Tumor necrosis factor; TNFi: Tumor necrosis factor inhibitor.

## Competing interests

The authors declare that they have no competing interests.

## Authors’ contributions

CID and SG made substantial contributions to the conception and design of the study, performed experiments, analyzed the results, made the figures and wrote the paper. TM made substantial contributions to the conception and design of the study, performed statistical analysis and was involved in drafting the manuscript and revising it critically for important intellectual content. BC, MH and JM made substantial contributions to the conception and design of the study and were involved in drafting the manuscript and revising it critically for important intellectual content. All authors read and approved the final manuscript.

## Supplementary Material

Additional file 1 Absolute values of B-cell subsets in patients and controls. Click here for file
