# Supplementary material for: High levels of memory B cells are associated with response to a first tumor necrosis factor inhibitor in patients with rheumatoid arthritis in a longitudinal prospective study
Source: Arthritis Res Ther. 2014 Apr 15;16(2):R95. doi: 10.1186/ar4543 (PMC4060280; doi:10.1186/ar4543)
Supplement: Additional file 1 — Absolute values of B-cell subsets in patients and controls. [file ar4543-S1.docx]

**Additional file 1. Absolute values of B-cell subsets in patients and controls**

| B-cell subsets (/mm^3^) | Controls  (n=11) | All RA patients  (n=72) | DMARD-naïve patients  (n=11) | p1 | p2 | TNFi-naïve patients  (n=34) | TNFi ongoing  (n=16) | p3 | Baseline TNFi introduction  (n=9) | | p4 |
| --- | --- | --- | --- | --- | --- | --- | --- | --- | --- | --- | --- |
|  |  |  |  |  |  |  |  |  | Baseline | 3 months |  |
| CD19^+^ | 158±30 | 91±10 | 111±30 | **0.02** | 0.27 | 97±12 | 97±19 | 0.98 | 100±56 | 144±83 | 0.13 |
| CD27^+^ | 50±9 | 23±3 | 23±8 | **<0.01** | **0.04** | 25±4 | 24±6 | 0.94 | 29±21 | 52±43 | 0.07 |
| CD27^+^IgD^+^ | 19±4 | 9±1 | 9±4 | **<0.01** | 0.09 | 10±2 | 10±3 | 0.94 | 12±11 | 23±25 | 0.21 |
| CD27^+^IgD^-^ | 33±7 | 17±2 | 16±7 | **<0.01** | 0.09 | 17±3 | 16±4 | 0.83 | 17±12 | 31±26 | 0.06 |
| CD27^-^IgD^+^ | 108±25 | 67±8 | 84±24 | 0.12 | 0.48 | 66±10 | 67±15 | 0.95 | 64±36 | 82±44 | 0.32 |
| CD27^-^IgD^-^ | 7±2 | 4±1 | 3±2 | **0.01** | 0.17 | 4±1 | 5±1 | 0.90 | 5±3 | 8±4 | 0.30 |
| CD24^hi^CD38^hi^ | 14±3 | 8±1 | 9±3 | 0.15 | 0.20 | 7±2 | 8±3 | 0.82 | 9±8 | 9±7 | 0.30 |
| CD24^hi^CD27^+^ | 37±8 | 16±3 | 15±8 | **<0.01** | 0.08 | 18±3 | 22±6 | 0.53 | 20±20 | 42±40 | 0.08 |
| CD5^+^ | 20±5 | 19±4 | 14±5 | 0.22 | 0.46 | 14±3 | 12±5 | 0.73 | 16±14 | 20±27 | 0.81 |

CD27^+^ memory B cells, CD27^+^IgD^+^ pre-switch memory B cells, CD27^+^IgD^-^ post-switch memory B cells, CD27^-^IgD^+^ naïve B cells, CD27^-^IgD^-^ double-negative B cells, CD38^hi^ plasmablasts. All values are expressed in adjusted mean±SD. p1, p-value comparing controls and all RA patients; p2, p-value comparing controls and DMARD-naïve patients. p3, p-value comparing TNFi-naïve and TNFi ongoing (currently taking TNFi agent). P-values were adjusted for age, gender and steroid dose. p4, p-value comparing baseline and 3-month data for patients with TNFi introduced at baseline.
